# The role of adaptation current in synchronously firing inhibitory neural networks with various topologies

**DOI:** 10.1186/1471-2202-16-S1-P303

**Published:** 2015-12-18

**Authors:** Scott Rich, Victoria Booth, Michal Zochowski

**Affiliations:** 1 Department of Mathematics, University of Michigan, Ann Arbor, MI 48104, USA; 2 Department of Applied Physics, University of Michigan, Ann Arbor, MI 48104, USA

## 

Inhibitory neural networks have the capacity to fire synchronously depending strongly on synaptic current dynamics. Various types of inhibitory interneurons, including some with adaptation currents, are present in hippocampal circuits and are implicated in governing overall network pattern formation. However, the contribution of intrinsic cell firing patterns of interneurons to these patterns has not been fully investigated. Understanding how both synaptic and cellular properties contribute to the propensity for inhibitory neural networks to synchronize is thus an invaluable tool for investigating hippocampal network pattern formation. Through numerical simulation of large, spiking neuron, inhibitory networks, we investigate the role of the slow, adapting M-type K+ current in network pattern formation. This adapting current plays a role in hippocampal interneurons, including the OLM cells, in which the blockade of M-current by acetylcholine or other neurotransmitters switches the neuronal firing rate-current relation (f-I curve) from Type II to Type I. Other types of interneurons, such as fast-spiking PV cells, display Type II f-I curves without any adaptation current. Thus, we consider networks of three cell types: Type I neurons and Type II neurons that either contain [1] or do not contain an M-type K+ adaptation current. All cell types are modeled in the Hodgkin-Huxley formalism. Heterogeneity is introduced to the networks through randomized external applied current to the neurons. We vary network connection topologies using the Small World Network Paradigm [2] in order to systematically investigate the role of connectivity between local and random topologies.

**Figure 1:**
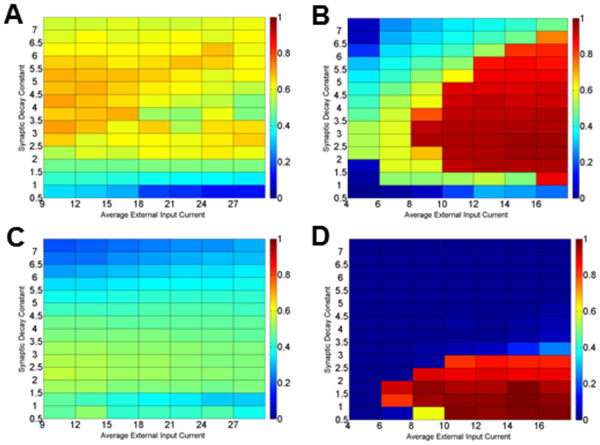
**Differential synchronization properties (scored by the Golomb measure **[3]) **of two inhibitory networks as a function of synaptic current duration, external drive and synaptic strength**. In 1000 neuron networks with 300 random incoming synapses per neuron, Type II networks without an adaptation current show clustering, resulting in a moderate synchronization measure, for nearly all parameters with low synaptic weight (A). Higher synaptic weight diminishes clustering (C). In identical simulations with low synaptic weight in Type II networks with an adaptation current, full synchronization is displayed when the synaptic decay constant is in an optimal range that grows with the external input current (B). Stronger synaptic weight reduces the synchronization parameter range towards shorter lasting synaptic currents (D).

To probe the interaction of cellular and synaptic properties influencing synchronization in these networks, we vary the time constant of decay of the inhibitory synaptic current and the intrinsic cellular frequencies by varying the mean of the distribution of external currents applied to each neuron. With sparse, nearest neighbor connection topologies, Type I networks exhibit stationary activity patterns reminiscent of standing waves while Type II neurons exhibit traveling wave activity patterns that sweep across the network. These traveling waves are much more robust and regular when the Type II neurons contain the adaptation current. With sparse random connectivity, Type II networks without an adaptation current exhibit cluster-firing patterns, in which cells segregate into multiple clusters that show synchrony within the cluster but not across clusters. In contrast, Type II networks with the adaptation current display full synchronization for some parameter ranges, but do not exhibit robust clustering (Figure 1). Preliminary analysis suggests that some of these results are due to the differences between Type I and Type II f-I profiles and that the adaptation current may enhance the effects of these differences.
